# The Neuroscience of Tinnitus: Understanding Abnormal and Normal Auditory Perception

**DOI:** 10.3389/fnsys.2012.00053

**Published:** 2012-07-11

**Authors:** Jos J. Eggermont, Larry E. Roberts

**Affiliations:** ^1^Department of Physiology and the Hotchkiss Brain Institute, University of CalgaryCalgary, AB, Canada; ^2^Department of Pharmacology and the Hotchkiss Brain Institute, University of CalgaryCalgary, AB, Canada; ^3^Department of Psychology and the Hotchkiss Brain Institute, University of CalgaryCalgary, AB, Canada; ^4^Department of Psychology, Neuroscience, and Behaviour, McMaster UniversityHamilton, ON, Canada

Tinnitus (chronic ringing of the ears in the absence of a sound source) is a major public health challenge affecting quality of life for millions of individuals around the world. Its principal cause (damage to the cochlea, which may be hidden and detected years after injury) appears to be increasing among youthful populations owing to exposure to recreational and occupational sounds for which current protective standards may be inadequate. And at present, there are no curative treatments for tinnitus. These facts alone, and the looming public health challenge they portend, are sufficient to spark its study. But research into the neural basis of tinnitus also addresses a fundamental question in neuroscience. If we can understand how the brain generates the sound of tinnitus, we may gain insight into the question of how the brain generates the sensation of other sounds. The papers published in this special issue (indicated in italics) address topics related to the neural basis of tinnitus, their implications for hearing, and the health challenge.

## Mechanisms Underlying Tinnitus

Deafferentation of central auditory structures by cochlear injury leads to several neural changes in auditory pathways that appear to underlie the sensation of tinnitus (discussed by *Brozoski et al., 2012; Diesch et al., 2012b; Langers et al., 2012; Middleton and Tzounopoulos, 2012; Schaette and Kempter, 2012; Stolzberg et al., 2012 and other papers*). Included among the neural changes are tonotopic map reorganization in auditory cortical and thalamic structures, hyperactivity in these structures (but typically not in auditory nerve fibers), increased burst firing in subcortical auditory nuclei, and increased synchronous neural activity particularly in tonotopic regions affected by hearing loss where tinnitus percepts also localize (Noreña and Eggermont, 2006; Roberts et al., 2010). Reduced input from the auditory periphery appears to trigger adaptive compensatory shifts in the balance of excitation and inhibition that may preserve neuron firing rates within a prescribed range; however an unwanted side effect reviewed by *Schaette and Kempter (2012)* may be an increase spontaneous neural activity that when phase locked into synchronous patterns leads to the experience of tinnitus percepts. Neural changes underlying tinnitus appear to modify the expression of training-induced neural plasticity in the primary (A1) but not secondary (A2) auditory cortex of human tinnitus sufferers, reflecting diminished inhibition and enhanced neural synchrony in regions of A1 affected by hearing loss (*Roberts et al., 2012*). Attentional effects on the auditory steady state response in tinnitus patients were deemed unlikely (*Diesch et al., 2012a*). Although cortical map reorganization cannot itself generate a tinnitus sound (only the activity of the affected neurons can do this), map reorganization is widely believed to play an enabling role in the generation of tinnitus. However, *Langers et al. (2012)* were unable to detect macroscopic map reorganization below 8 kHz in functional imaging data in human tinnitus patients with normal audiometric thresholds. Whether map reorganization can be detected at higher frequencies in such patients is not known but may be the case. Map reorganization assessed by neuromagnetic imaging has been reported in tinnitus patients for whom hearing loss was present (Wienbruch et al., 2006). Genetic aspects of tinnitus have so far not been conclusively demonstrated and the paper by *Sand et al. (2012)* follows that trend. An important mechanism in the induction of neural plasticity is stress. Stress may have protective effects against noise trauma, but a combination of stress and hearing loss could enhance the likelihood of tinnitus (*Mazurek et al., 2012*). The involvement of stress networks in tinnitus is reviewed in *Vanneste and De Ridder (2012)*.

Other papers in the special issue describe animal models and computational approaches to understand mechanisms of tinnitus. Animal models are important, because such models permit measurements and interventions that cannot be performed on human tinnitus subjects. In one animal model the presence of tinnitus is signaled by making tinnitus a cue for a behaviorally relevant event. *Brozoski et al. (2012)* combined this method with magnetic resonance spectroscopy to uncover alterations in GABAergic and glutaminergic neurotransmission in specific subcortical auditory nuclei in rats showing behavioral evidence of tinnitus after traumatic noise exposure. A second and more widely used approach introduced by Turner et al. (2006), cautioned by Eggermont (2012), and evaluated by *Dehmel et al. (2012)* determines whether a tinnitus sound (in this case induced by noise exposure in guinea pigs) fills a silent gap in a background sound that would otherwise suppress an evoked startle response. *Stolzberg et al. (2012)* and *Guitton (2012)* discuss in depth how neural changes induced by salicylate in animal preparations are both congruent and in some respects different from those observed when tinnitus and hearing loss are induced by noise exposure. *Middleton and Tzounopoulos (2012)* call for detailed investigations of network neural activity in animal models of tinnitus, looking specifically at communication between thalamic nuclei and brain regions known to be active in tinnitus. Taking a different tack, *Schaette and Kempter (2012)* discuss how computational studies can reveal (or refute) whether neural network models of tinnitus are able to generate properties of tinnitus revealed in physiological and psychoacoustic studies. They emphasize that incorporating forms of neural plasticity in the models determines whether the models are able to simulate measured attributes of tinnitus.

## Tinnitus and Hearing

An important fact about tinnitus revealed by functional brain imaging studies is that the brain regions affected by tinnitus extend beyond auditory structures to include brain areas that are involved in higher level cognitive processing. *Langguth et al. (2012)* give a concise description of the brain areas that distinguish between individuals with and without tinnitus. Strikingly, the affected structures (which include subdivisions of prefrontal cortex, parietal cortex, the cingulate gyrus, and the insula) are similar to brain regions that show augmented BOLD responses during performance on attention-demanding cognitive tasks in normal hearing individuals. Evidence from neurocognitive research reviewed elsewhere by Dehaene and Changeux (2011) supports the view that activation of this network (called the Global Neuronal Workspace by Dehaene and Changeux, 2011, adapted from Baars, 1989) is closely correlated with the experience of conscious awareness. Because tinnitus is a persisting conscious percept it is perhaps not surprising that functional imaging of tinnitus has revealed similar global network activity. Correspondingly, it has been suggested by many researchers that aberrant neural activity restricted to auditory pathways is not sufficient for the experience of tinnitus, but that global network activity must be engaged (Schlee et al., 2009; De Ridder et al., 2011). It has also been proposed that different tinnitus attributes may reflect the activity of specialized nodes within this network (see *Leaver et al., 2012; Vanneste and De Ridder, 2012*) and that communication within and among the nodes may explain documented oscillatory correlates of tinnitus in the delta, alpha, and gamma bands (*Middleton and Tzounopoulos, 2012*). Building on the network concept, *Elgoyhen et al. (2012)* propose that drugs that have multiple low level effects on synaptic processes in highly specialized pathways (therapeutic “shotguns”) may prove to be more effective at disrupting network behavior and reducing tinnitus than drugs aimed at specific triggering mechanisms. *Brozoski et al. (2012)* similarly suggest in this issue that drugs targeting GABAergic as well as glutaminergic function may be more effective in reducing tinnitus than pharmaceuticals that have more specific action profiles.

One omission in the tinnitus literature (current papers not excepted) is a discussion of the possible role of the basal forebrain cholinergic system in the triggering and maintaining network behavior in chronic tinnitus. Cholinergic efferents originating from several nuclei in the basal forebrain project to all regions of the neocortical mantle in a coarse regional topography (Jiménez-Capdeville et al., 1997; Sarter et al., 2009), including prefrontal, parietal, and allocortical structures comprising the Global Neuronal Workspace of Dehaene and Changeux (2011). These projections make the targeted pyramidal neurons more sensitive to their afferent inputs by promoting the extrasynaptic release of acetylcholine or by acting on heteroreceptors to achieve function-specific effects (Sarter et al., 2009). A parallel GABAergic innervation has been described (Freund and Meskenaite, 1992) targeting inhibitory cortical interneurons suggesting a synergistic effect. The basal forebrain system is known to gate neural plasticity in the cortex of mature animals induced by sounds that signal behaviorally important goals (Ramanathan et al., 2009). In tinnitus the disparity that exists between what the brain thinks it is hearing (this expectation coded by synchronous activity in cortical regions affected by hearing loss) and thalamocortical input arriving from the damaged ear could engage the basal forebrain system as the brain attempts (unsuccessfully) to construct a more accurate central representation of the auditory scene.

## The Health Challenge

Several papers in the current issue underscore the difficulty of effectively treating chronic tinnitus sounds. *Adamchic et al. (2012)* present evidence suggesting a long-lasting and cumulative benefit for tinnitus of coordinated-reset sound therapy and a possible long-lasting desynchronizing effect on pathological, tinnitus-related neuronal synchrony. *Kreuzer et al. (2011)* investigated whether disrupting both auditory and non-auditory hubs in the tinnitus network with repetitive transcranial magnetic stimulation (rTMS) gave greater therapeutic benefit than disrupting auditory regions alone. A modest reduction of tinnitus handicap scores was found after rTMS treatment in a subset of patients, in agreement with previous studies of rTMS therapy. However, the combined protocol while trending toward greater improvement was not significantly more effective. Notably, handicap scores improved significantly between two baseline measurements that were taken before rTMS treatment had begun. This finding suggests that improvements after an intake assessment may be spuriously interpreted as treatment effects if baseline stability is not assessed (see *Lehner et al., 2012* for further discussion). None of the studies described herein reported results from psychoacoustic measurements of tinnitus, which have been found to be more resistant to change in the treatment literature (Roberts and Bosnyak, 2010). However, decreases in tinnitus distress are often reported after sound or rTMS therapy, and the value of such decreases for individual patients should not be overlooked. *Searchfield et al. (2012)* propose a broad framework for understanding and managing tinnitus based on Helson's Adaptation Level Theory (Helson, 1964). It is hoped that the framework will encourage greater empirical investigation of factors that affect tinnitus audibility (attention, context, and personality) and the outcome of sound therapies.

Taking a different approach, *Pantev et al. (2012)* describe their research which found that listening to music with frequencies in the tinnitus region notched out reduced electrophysiological correlates of tinnitus accompanied by a reduction in tinnitus loudness assessed by a visual analog scale. They propose that lateral inhibition distributed to the tinnitus frequencies may underlie this result. A subsequent short-term application of the sound therapy observed success only for patients with a dominant tinnitus frequency of less than 8 kHz. Cochlear implant patients provide an opportunity to assess the effect on tinnitus of restoring input to auditory pathways (*Chang and Zeng, 2012*). Nine of the 13 patients (69%) reported a decrease in tinnitus when the implant was switched on, and in five of these cases tinnitus suppression was complete or near complete. Whether suppression persists after CI stimulation was not systematically assessed although one patient reported a persisting benefit 24 h later. Notably, tinnitus suppression was better in this study when the implant was programmed specifically for tinnitus suppression and not for optimal speech processing.

While hearing loss measured by the audiogram is present in the majority of cases of chronic tinnitus, audiometric threshold shifts are not always seen, and such shifts can occur in the absence of tinnitus (Roberts et al., 2008). Improved measures of cochlear function beyond those dependent on threshold responses are needed to understand these disparities and characterize with greater precision the environmental conditions that pose risks for cochlear injury. The question is important. Almost 20% of American adolescents show changes in their audiograms indicative of hearing loss related to noise exposure (Shargorodsky et al., 2010), and the degree of threshold shift that sets the stage for tinnitus does not appear to be large (Wienbruch et al., 2006). Cochlear damage expressed initially in high threshold auditory nerve fibers appears to be progressive and may not express until later in life (Kujawa and Liberman, 2006, 2009) when age-related declines add to risks of tinnitus and impaired hearing function.

Research on tinnitus has also sparked a new and important interest in understanding how long term passive exposure to background sound modifies central auditory processing in the mature brain. Contrary to the view that behavioral relevance is a prerequisite for modifying neural representations in adults (Keuroghlian and Knudsen, 2007), recent research has shown that passive exposure to background sounds at low levels can have profound effects on auditory cortical processing (Noreña et al., 2006; Pienkowski and Eggermont, 2009). Sound therapies for tinnitus are based on this principle, and while these therapies may in suitable circumstances deliver beneficial results (Davis et al., 2008; Roberts and Bosnyak, 2010), foundational knowledge of the enabling conditions and the mechanisms at work is lacking. The relevance of this topic extends well beyond tinnitus. In animal studies chronic exposure to background sound that resembles many human workplace environments produces substantial changes in central auditory processing that can lead to impaired performance on auditory tasks, even when conventional threshold measures of cochlear function are in the normal range (Pienkowski and Eggermont, 2012; Zhou and Merzenich, 2012). Knowledge of the processes involved will help understand the risks for central and peripheral hearing as well as potential benefits for remediation and prevention of hearing disorder.

## References

[B1] Adamchic I. Hauptmann C. Tass P. A. (2012). Changes of oscillatory activity in pitch processing network and related tinnitus relief induced by acoustic CR neuromodulation. Front. Syst. Neurosci. 6:18. 10.3389/fnsys.2012.00018 22493570 PMC3319974

[B2] Baars B. J. (1989). A Cognitive Theory of Consciousness. Cambridge, MA: Cambridge University Press.

[B3] Brozoski T. Odintsov B. Bauer C. (2012). Gamma-aminobutyric acid and glutamic acid levels in the auditory pathway of rats with chronic tinnitus: a direct determination using high resolution point-resolved proton magnetic resonance spectroscopy (1H-MRS). Front. Syst. Neurosci. 6:9. 10.3389/fnsys.2012.00009 22383901 PMC3285819

[B4] Chang J. E. Zeng F. (2012). Tinnitus suppression by electric stimulation of the auditory nerve. Front. Syst. Neurosci. 6:19. 10.3389/fnsys.2012.00019 22479238 PMC3315113

[B5] Davis P. B. Wilde R. A. Steed L. G. Hanley P. J. (2008). Treatment of tinnitus with a customized acoustic neural stimulus: a controlled clinical study. Ear Nose Throat J. 87, 330–339. 18561116

[B6] De Ridder D. Elgoyhen A. B. Romo R. Langguth B. (2011). Phantom percepts: tinnitus and pain as persisting aversive memory networks. Proc. Natl. Acad. Sci. U.S.A. 108, 8075–8080. 10.1073/pnas.1018466108 21502503 PMC3100980

[B7] Dehaene S. Changeux J.-P. (2011). Experimental and theoretical approaches to conscious processing. Neuron 70, 200–227. 10.1016/j.neuron.2011.03.018 21521609

[B8] Dehmel S. Eisinger D. Shore S. E. (2012). Gap prepulse inhibition and auditory brainstem-evoked potentials as objective measures for tinnitus in guinea pigs. Front. Syst. Neurosci. 6:42. 10.3389/fnsys.2012.00042 22666193 PMC3364697

[B9] Diesch E. Andermann M. Rupp A. (2012a). Is the effect of tinnitus on auditory steady-state response amplitude mediated by attention? Front. Syst. Neurosci. 6:38 10.3389/fnsys.2012.00038 22661932 PMC3357113

[B10] Diesch E. Schummer V. Kramer M. Rupp A. (2012b). Structural changes of the corpus callosum in tinnitus. Front. Syst. Neurosci. 6:17 10.3389/fnsys.2012.00017 22470322 PMC3312098

[B11] Eggermont J. J. (2012). Hearing loss, hyperacusis, and tinnitus: what is modeled in animal research? Hear. Res.. 10.1016/j.heares.2012.01.005 22330978

[B12] Elgoyhen A. B. Langguth B. Vanneste S. De Ridder D. (2012). Tinnitus: network pathophysiology-network pharmacology. Front. Syst. Neurosci. 6:1. 10.3389/fnsys.2012.00001 22291622 PMC3265967

[B13] Freund T. F. Meskenaite V. (1992). Gamma-aminobutyric acid-containing basal forebrain neurons innervate inhibitory interneurons in the neocortex. Proc. Natl. Acad. Sci. U.S.A. 89, 738–742. 10.1073/pnas.89.2.738 1731348 PMC48314

[B14] Guitton M. J. (2012). Tinnitus: pathology of synaptic plasticity at the cellular and system levels. Front. Syst. Neurosci. 6:12. 10.3389/fnsys.2012.00012 22408611 PMC3297194

[B15] Helson H. (1964). Adaptation-Level Theory. New York: Harper and Row.

[B16] Jiménez-Capdeville M. E. Dykes R. W. Myasnikov A. A. (1997). Differential control of cortical activity by the basal forebrain in rats: a role for both cholinergic and inhibitory influences. J. Comp. Neurol. 381, 53–67 10.1002/(SICI)1096-9861(19970428)381:1<53::AID-CNE5>3.3.CO;2-L 9087419

[B17] Keuroghlian A. S. Knudsen E. I. (2007). Adaptive auditory plasticity in developing and adult animals. Prog. Neurobiol. 82, 109–121. 10.1016/j.pneurobio.2007.03.005 17493738

[B18] Kreuzer P. M. Landgrebe M. Schecklmann M. Poeppl T. B. Vielsmeier V. Hajak G. Kleinjung T. Langguth B. (2011). Can temporal repetitive transcranial magnetic stimulation be enhanced by targeting affective components of tinnitus with frontal rTMS? A randomized controlled pilot trial. Front. Syst. Neurosci. 5:88. 10.3389/fnsys.2011.00088 22069382 PMC3208342

[B19] Kujawa S. G. Liberman M. C. (2006). Acceleration of age-related hearing loss by early noise exposure: evidence of a misspent youth. J. Neurosci. 26, 2115–2123. 10.1523/JNEUROSCI.4985-05.2006 16481444 PMC1855187

[B20] Kujawa S. G. Liberman M. C. (2009). Adding insult to injury: cochlear nerve degeneration after “temporary” noise-induced hearing loss. J. Neurosci. 29, 14077–14085. 10.1523/JNEUROSCI.2845-09.2009 19906956 PMC2812055

[B21] Langers D. R. M. de Kleine E. van Dijk P. (2012). Tinnitus does not require macroscopic tonotopic map reorganization. Front. Syst. Neurosci. 6:2. 10.3389/fnsys.2012.00002 22347171 PMC3269775

[B22] Langguth B. Schecklmann M. Lehner A. Landgrebe M. Poeppl T. B. Kreuzer P. M. Schlee W. Weisz N. Vanneste S. De Ridder D. (2012). Neuroimaging and neuromodulation: complementary approaches for identifying the neuronal correlates of tinnitus. Front. Syst. Neurosci. 6:15. 10.3389/fnsys.2012.00015 22509155 PMC3321434

[B23] Leaver A. M. Seydell-Greenwald A. Turesky T. K. Morgan S. Kim H. J. Rauschecker J. P. (2012). Cortico-limbic morphology separates tinnitus from tinnitus distress. Front. Syst. Neurosci. 6:21. 10.3389/fnsys.2012.00021 22493571 PMC3319920

[B24] Lehner A. Schecklmann M. Landgrebe M. Kreuzer P. M. Poeppl T. B. Frank E. Vielsmeier V. Kleinjung T. Rupprecht R. Langguth B. (2012). Predictors for rTMS response in chronic tinnitus. Front. Syst. Neurosci. 6:11. 10.3389/fnsys.2012.00011 22383902 PMC3284861

[B25] Mazurek B. Haupt H. Olze H. Szczepek A. J. (2012). Stress and tinnitus—from bedside to bench and back. Front. Syst. Neurosci. 6:47. 10.3389/fnsys.2012.00047 22701404 PMC3371598

[B26] Middleton J. W. Tzounopoulos T. (2012). Imaging the neural correlates of tinnitus: a comparison between animal models and human studies. Front. Syst. Neurosci. 6:35. 10.3389/fnsys.2012.00035 22586378 PMC3343475

[B27] Noreña A. J. Eggermont J. J. (2006). Enriched acoustic environment after noise trauma abolishes neural signs of tinnitus. Neuroreport 17, 559–563 10.1097/00001756-200604240-00001 16603911

[B28] Noreña A. J. Gourévitch B. Aizawa N. Eggermont J. J. (2006). Spectrally enhanced acoustic environment disrupts frequency representation in cat auditory cortex. Nat. Neurosci. 9, 932–939 10.1038/nn0906-1193a 16783369

[B29] Pantev C. Okamoto H. Teismann H. (2012). Music-induced cortical plasticity and lateral inhibition in the human auditory cortex as foundations for tonal tinnitus treatment. Front. Syst. Neurosci. 6:50. 10.3389/fnsys.2012.00050 22754508 PMC3384223

[B30] Pienkowski M. Eggermont J. J. (2009). Long-term, partially-reversible reorganization of frequency tuning in mature cat primary auditory cortex can be induced by passive exposure to moderate-level sounds. Hear. Res. 257, 24–40. 10.1016/j.heares.2009.07.011 19647789

[B31] Pienkowski M. Eggermont J. J. (2012). Reversible long-term changes in auditory processing in mature auditory cortex in the absence of hearing loss induced by passive, moderate-level sound exposure. Ear Hear. 33, 305–314. 10.1093/eurheartj/ehr366 22343545

[B32] Ramanathan D. Tuszynski M. H. Conner J. M. (2009). The basal forebrain cholinergic system is required specifically for behaviorally mediated cortical map plasticity. J. Neurosci. 29, 5992–6000. 10.1523/JNEUROSCI.0230-09.2009 19420265 PMC6665226

[B33] Roberts L. E. Bosnyak D. J. (2010). “Auditory training in tinnitus,” in Textbook of Tinnitus, eds Møller A. Kleinjung T. Langguth B. De Ridder D. (New York: Humana-Springer) 563–574.

[B34] Roberts L. E. Bosnyak D. J. Thompson D. C. (2012). Neural plasticity expressed in central auditory structures with and without tinnitus. Front. Syst. Neurosci. 6:40. 10.3389/fnsys.2012.00040 22654738 PMC3361130

[B35] Roberts L. E. Eggermont J. J. Caspary D. C. Shore S. E. Melcher J. R. Kaltenbach J. A. (2010). Ringing ears: the neuroscience of tinnitus. J. Neurosci. 30, 14980–14986. 10.1523/JNEUROSCI.4283-10.2010 21068300 PMC3073522

[B36] Roberts L. E. Moffat G. Baumann M. Ward L. M. Bosnyak D. J. (2008). Residual inhibition functions overlap tinnitus spectra and the region of auditory threshold shift. J. Assoc. Res. Otolaryngol. 9, 417–435. 10.1007/s10162-008-0136-9 18712566 PMC2580805

[B37] Sand P. G. Langguth B. Itzhacki J. Bauer A. Geis S. Cárdenas-Conejo Z. E. Pimentel V. Kleinjung T. (2012). Resequencing of the auxiliary GABAB receptor subunit gene KCTD12 in chronic tinnitus. Front. Syst. Neurosci. 6:41. 10.3389/fnsys.2012.00041 22654739 PMC3360237

[B38] Sarter M. Parikh V. Howe W. M. (2009). nAChR agonist-induced cognition enhancement: integration of cognitive and neuronal mechanisms. Biochem. Pharmacol. 78, 658–667. 10.1016/j.bcp.2009.04.019 19406107 PMC2750036

[B39] Schaette R. Kempter R. (2012). Computational models of neurophysiological correlates of tinnitus. Front. Syst. Neurosci. 6:34. 10.3389/fnsys.2012.00034 22586377 PMC3347476

[B40] Schlee W. Mueller N. Hartmann T. Keil J. Lorenz I. Weisz N. (2009). Mapping cortical hubs in tinnitus. BMC Biol. 7, 80. 10.1186/1741-7007-7-80 19930625 PMC2787501

[B41] Searchfield G. D. Kobayashi K. Sanders M. (2012). An adaptation level theory of tinnitus audibility. Front. Syst. Neurosci. 6:46. 10.3389/fnsys.2012.00046 22707935 PMC3374480

[B42] Shargorodsky J. Curhan S. G. Curhan G. C. Eavey R. (2010). Change in prevalence of hearing loss in US adolescents. JAMA 304, 772–778. 10.1001/jama.2010.1124 20716740

[B43] Stolzberg D. Salvi R. J. Allman B. L. (2012). Salicylate toxicity model of tinnitus. Front. Syst. Neurosci. 6:28. 10.3389/fnsys.2012.00028 22557950 PMC3341117

[B44] Turner J. G. Brozoski T. J. Bauer C. A. Parrish J. L. Myers K. Hughes L. F. Caspary D. M. (2006). Gap detection deficits in rats with tinnitus: a potential novel screening tool. Behav. Neurosci. 120, 188–195. 10.1037/0735-7044.120.1.188 16492129

[B45] Vanneste S. De Ridder D. (2012). The auditory and non-auditory brain areas involved in tinnitus. An emergent property of multiple parallel overlapping subnetworks. Front. Syst. Neurosci. 6:31. 10.3389/fnsys.2012.00031 22586375 PMC3347475

[B46] Wienbruch C. Paul I. Weisz N. Elbert T. Roberts L. E. (2006). Frequency organization of the 40-Hz auditory steady-state response in normal hearing and in tinnitus. Neuroimage 33, 180–194. 10.1016/j.neuroimage.2006.06.023 16901722

[B47] Zhou X. Merzenich M. M. (2012). Environmental noise exposure degrades normal listening processes. Nat. Commun. 3, 843. 10.1038/ncomms1849 22588305

